# Olopatadine Exerts Multifaceted Protective Effects in an Ovalbumin-Induced Allergic Eye Disease Mouse Model

**DOI:** 10.3390/bioengineering13091060

**Published:** 2026-09-12

**Authors:** Basanta Bhujel, Kyu Sang Eah, Soon Suk Kang, Ho Seok Chung, Hun Lee, Jae-Yong Kim

**Affiliations:** 1 Department of Ophthalmology, Asan Medical Center, College of Medicine, University of Ulsan, Seoul 05505, Republic of Korea; basantabhujel86@gmail.com (B.B.); kseah0124@gmail.com (K.S.E.); joy_sskang@naver.com (S.S.K.);; 2 Department of Medical Science, University of Ulsan Graduate School, Seoul 05505, Republic of Korea

**Keywords:** allergic eye disease, allergic conjunctivitis, olopatadine, ovalbumin, inflammation

## Abstract

Allergic eye disease (AED), including allergic conjunctivitis, is a prevalent immune-mediated disorder of the ocular surface characterized by itching, redness, tearing, and ocular discomfort. Although olopatadine (OLO) is widely used as an antihistamine and mast cell stabilizer, its broader effects on immune-mediated inflammation, neuropeptide-associated responses, and epithelial–mucosal changes in AED remain incompletely understood. This study investigated the multifaceted effects of OLO in an ovalbumin (OVA)-induced AED mouse model. AED was induced in BALB/c mice by OVA sensitization and topical challenge, followed by OLO treatment during the challenge period. Clinical severity, scratching behavior, serum IgE, inflammatory cell infiltration (PMNs), mast cell degranulation, cytokine expression (IL-4, IL-5, IL-6, TNF-α, and IL-1β), macrophage infiltration (F4/80), iNOS expression, TRPV1, SP, and CGRP expression, ZO-1 expression, goblet cell density, mucin expression (MUC5AC), dysregulated cell proliferation (Ki67), and apoptotic cell death (TUNEL) were assessed. OVA challenge induced marked allergic responses, including elevated clinical scores, increased scratching behavior, elevated serum IgE, mast cell degranulation, inflammatory cell infiltration, and upregulation of Th2- and pro-inflammatory cytokines. In addition, OLO reduced TRPV1, SP, and CGRP expression, preserved ZO-1 expression and goblet cell density, and reduced excessive epithelial cell proliferation. Notably, OLO demonstrated multifaceted protective effects in AED, providing preclinical insights into its effects in experimental AED while acknowledging its established clinical use in humans.

## 1. Introduction

Allergic eye disease (AED), including allergic conjunctivitis, is among the most prevalent immune-mediated disorders affecting the ocular surface, imposing a substantial burden on patients and healthcare systems worldwide [1,2]. These conditions, which include seasonal and perennial allergic conjunctivitis, are characterized by symptoms such as itching, redness, tearing, and swelling, often accompanied by ocular discomfort [3]. While traditionally considered mild and transient, increasing evidence suggests that AED can become chronic and debilitating, particularly when inadequately controlled [4]. The persistence of symptoms and their impact on daily activities underscore the need for a deeper understanding of disease mechanisms and more effective management strategies [4].

The pathogenesis of AED is primarily driven by immunoglobulin E (IgE)-mediated hypersensitivity reactions initiated upon exposure to environmental allergens [2,5]. During the sensitization phase, antigen-presenting cells activate T helper 2 (Th2) lymphocytes, which secrete cytokines such as interleukin (IL)-4, IL-5, and IL-13. These cytokines promote B-cell class switching to IgE and facilitate mast cell sensitization, thereby priming the immune system for exaggerated responses upon re-exposure to allergens [1,5].

Subsequent allergen challenge leads to cross-linking of IgE bound to high-affinity immunoglobulin E receptors (FcεRI) on mast cells, triggering rapid degranulation and the release of histamine and other pro-inflammatory mediators. This early-phase response manifests clinically as conjunctival hyperemia, chemosis, tearing, and pruritus [6,7]. In addition, mast cell activation initiates a cascade of downstream signaling events that recruit inflammatory cells, including polymorphonuclear leukocytes (PMNs), contributing to the late-phase response. This sustained inflammatory state is further amplified by elevated levels of cytokines such as tumor necrosis factor-α (TNF-α) and interleukin-1β (IL-1β), which exacerbate allergic inflammation and vascular permeability [5,6].

Beyond immune activation, emerging evidence suggests that structural components such as the conjunctival epithelial barrier may influence disease progression by regulating allergen penetration and local inflammatory responses [8]. Moreover, inflammatory mediators can interact with sensory nerve endings, potentially contributing to pruritus and ocular discomfort and indicating an interaction between inflammation and sensory neural responses [9].

Despite the availability of various treatment options, including antihistamines, mast cell stabilizers, and anti-inflammatory agents, significant unmet needs remain in the management of AED. Most current treatments primarily target early-phase histamine-mediated responses and provide only partial relief, particularly in chronic or severe cases [2,10]. They often fail to adequately control late-phase inflammation, do not fully address ocular discomfort, and have a limited impact on underlying tissue alterations such as epithelial disruption [11,12]. These limitations highlight the need for more comprehensive treatment approaches that address multiple aspects of AED pathogenesis.

Olopatadine (OLO) is a well-established dual-acting agent with both antihistaminic and mast cell-stabilizing properties, making it a widely used treatment for AED [13,14,15]. By inhibiting histamine receptor activation and preventing mast cell degranulation, it effectively reduces key features of the early allergic response. In addition to these actions, OLO has been suggested to exert broader anti-inflammatory effects, potentially influencing cytokine production and cellular infiltration [2,4,10]. Although the antihistaminic and mast cell-stabilizing effects of OLO are well established, whether OLO influences neuropeptide-associated responses and conjunctival epithelial–mucosal changes in AED remains incompletely understood.

The ovalbumin (OVA)-induced AED model is a well-established experimental system that replicates key features of human allergic conjunctivitis [16,17,18]. OVA, a highly immunogenic protein, is used to sensitize and subsequently challenge animals, leading to a reproducible Th2-mediated immune response characterized by mast cell activation, eosinophil infiltration, and cytokine release [19,20]. This model closely mimics both the early and late phases of allergic inflammation and allows assessment of behavioral indicators of ocular discomfort [21]. Its reliability and relevance make it a useful experimental model for evaluating the protective effects of anti-allergic agents such as OLO.

**Figure 1:**
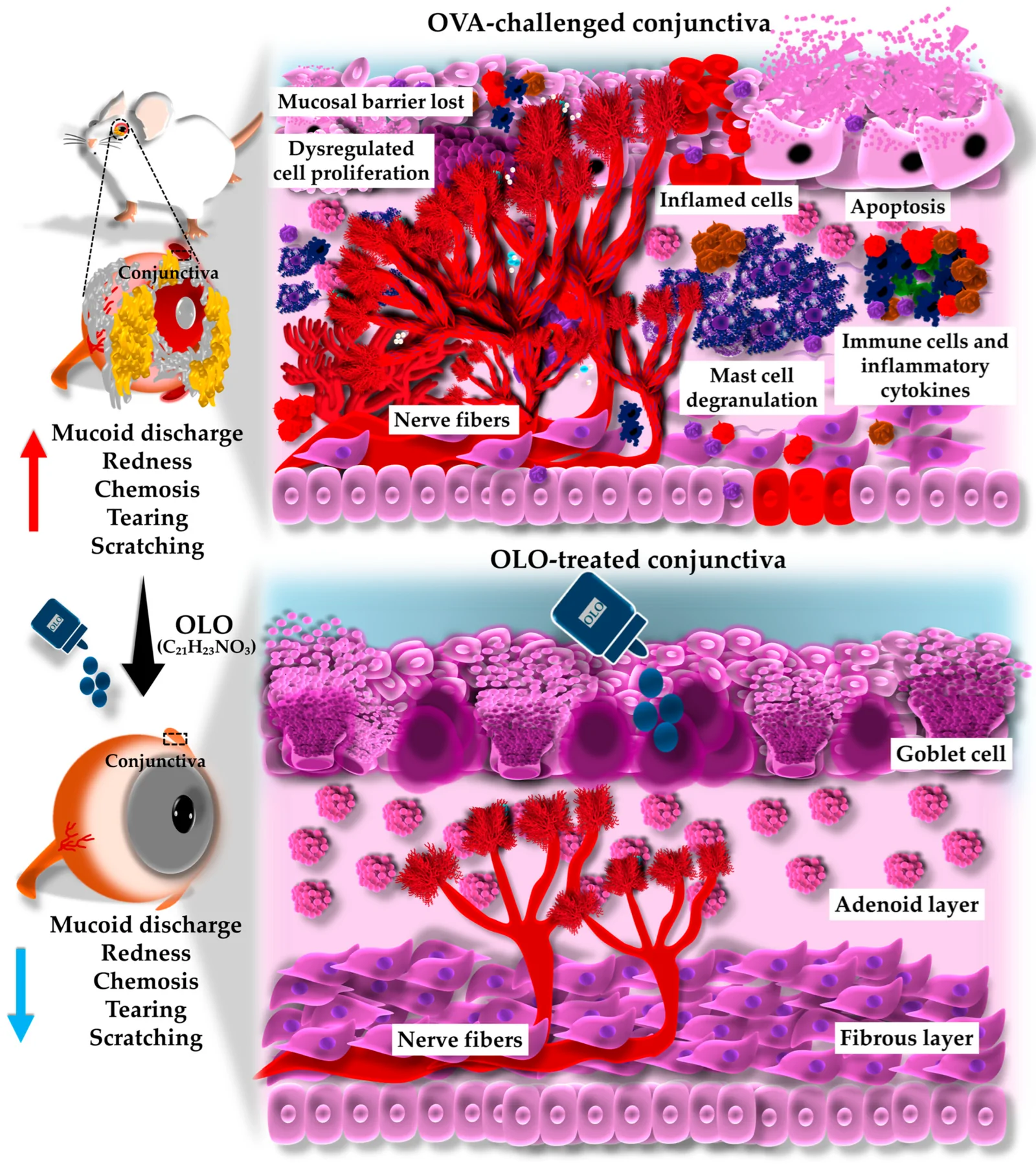
Schematic illustration of the protective effects of OLO in an OVA-induced experimental AED mouse model. OVA challenge induces characteristic clinical manifestations of AED, including conjunctival redness, chemosis, tearing, mucoid discharge, and increased scratching behavior. Within the conjunctiva, OVA exposure promotes mast cell degranulation, infiltration of inflammatory cells and pro-inflammatory cytokines, apoptosis, increased epithelial cell proliferation, and goblet cell loss, resulting in ocular surface alterations. In contrast, OLO treatment attenuates these allergic changes, reducing conjunctival redness, chemosis, tearing, mucoid discharge, and scratching behavior. In the conjunctiva, OLO reduces mast cell degranulation, inflammatory cell infiltration, and pro-inflammatory cytokine expression, while decreasing apoptosis and excessive epithelial cell proliferation and preserving goblet cells, thereby maintaining ocular surface features.

The present study was designed to evaluate the multifaceted protective effects of OLO across multiple disease-associated features of AED within a single OVA-induced mouse model. We assessed inflammatory responses, neuropeptide-associated marker expression, and epithelial–mucosal changes, providing an integrated assessment of the effects of OLO in experimental AED (Figure 1).

## 2. Materials and Methods

### 2.1. Animals

All procedures were performed in strict accordance with institutional and international guidelines for animal research and were approved by the Institutional Animal Care and Use Committee (IACUC) of the University of Ulsan and the Life Sciences Institute of Asan Medical Center. Experiments adhered to the ARVO Statement for the Use of Animals in Ophthalmic and Vision Research.

Twenty-one female BALB/C mice (8 weeks old; Orient Bio Inc., Seongnam, Republic of Korea) were acclimatized for one week under controlled conditions (12 h light/dark cycle) with ad libitum access to sterilized food and water. Animals were maintained in a specific pathogen-free facility, and all efforts were made to minimize distress.

### 2.2. Induction of AED

The AED mouse model was established using a standard OVA sensitization protocol [22]. Briefly, mice designated for sensitization received a single intraperitoneal (IP) injection of OVA (10 μg), aluminum hydroxide (ALUM; 1 mg), and pertussis toxin (PT; 300 ng) on day 0. After a 14-day sensitization period, animals were randomly divided into three groups (*n* = 7 each): normal, OVA, and OVA + OLO-treatment.

**Figure 2:**
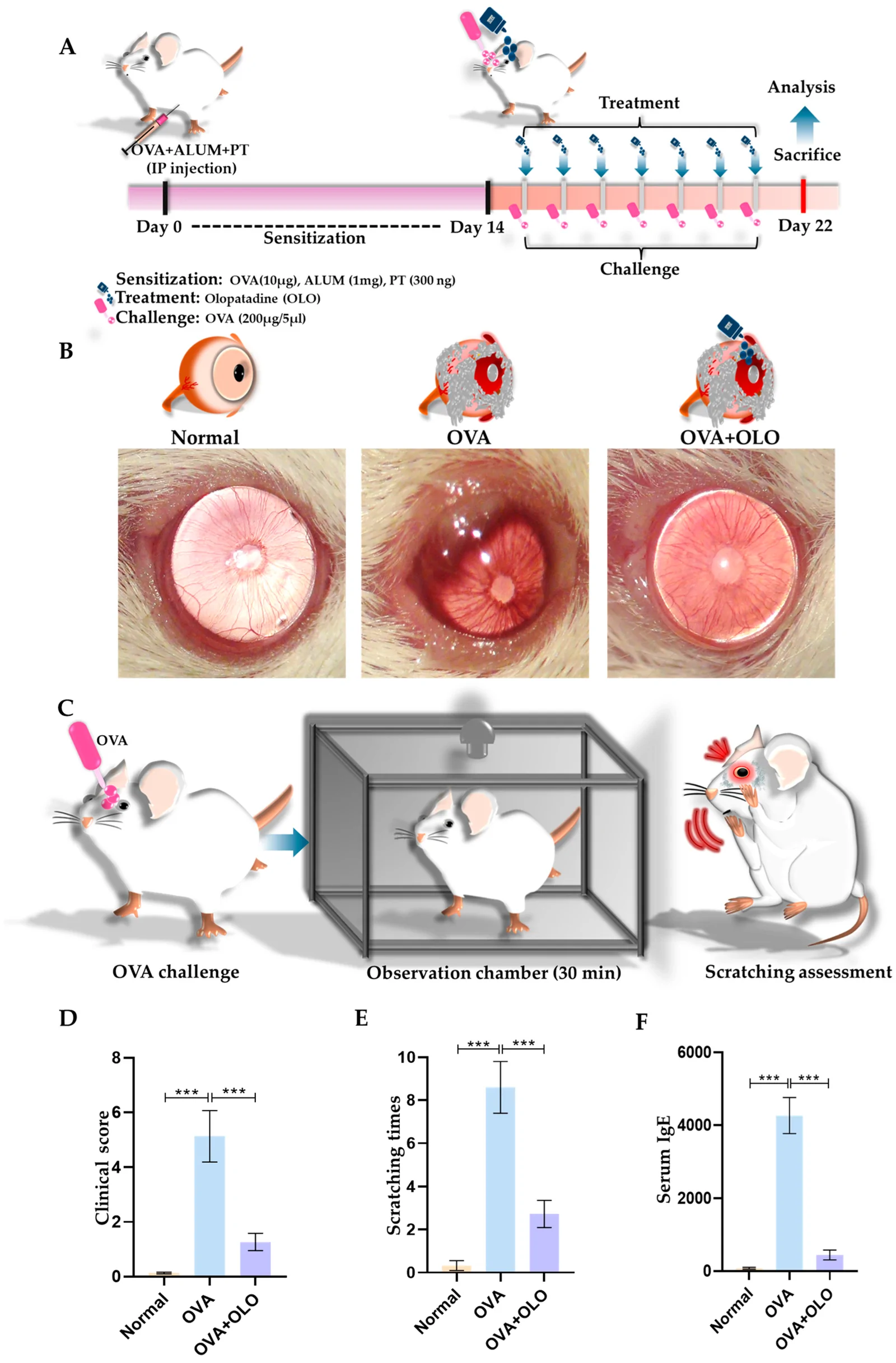
Establishment of the OVA-induced AED mouse model and evaluation of the protective effects of OLO: (**A**) Schematic representation of the experimental design and timeline. Mice were sensitized by IP injection of OVA, ALUM, and PT on day 0, followed by topical OVA challenges and OLO treatment from days 15–21. Samples were collected for analysis on day 22. (**B**) Representative slit lamp images of the ocular surface. (**C**) Diagrammatic representation of scratching behavior in the mice. Following topical administration of 200 μg/5 μL of OVA solution to the eye, mice were individually placed in an observation chamber. Hindlimb scratching directed toward the periocular region was recorded for 30 min, and the total number of scratching episodes was quantified as an indicator of allergic ocular discomfort. (**D**) Quantitative assessment of clinical score. (**E**) Quantitative analysis of scratching behavior. (**F**) Changes in the serum IgE concentration level. Scale bar = 1 mm. Mean ± SEM (*n* = 7). One-way ANOVA followed by Tukey’s post hoc test. *** *p*  <  0.001.

From days 15 to 21, mice in the OVA and OVA + OLO groups received topical OVA challenges (200 μg/5 μL) twice daily, while mice in the OVA + OLO group additionally received topical 0.1% OLO twice daily. The 0.1% OLO concentration was selected based on its use in previous experimental murine studies and its established use in topical ophthalmic formulations for allergic conjunctivitis [23,24]. The normal group received neither OVA sensitization nor topical treatment (Figure 2A). Mice were euthanized on day 22 via CO_2_ inhalation, and the left eyes were collected for the analyses.

### 2.3. Evaluation of Corneal Clinical Score

Ocular manifestations of AED were evaluated using a slit-lamp microscope. Clinical assessments were performed by a blinded observer and included conjunctival hyperemia, conjunctival chemosis, eyelid edema, and ocular discharge. Each parameter was scored on a scale of 0–3 according to previously established criteria [25]. The total clinical score was calculated as the sum of the four individual parameters, yielding a cumulative score ranging from 0 to 12, with higher scores indicating greater disease severity. Clinical assessments were performed at the final experimental time point.

### 2.4. Scratching Behavior

Animals were acclimated for 10 min before OVA was instilled onto the ocular surface. Periocular scratching was then recorded for 30 min and quantified as rapid hindlimb movements directed toward the eye (Figure 2C) [26].

### 2.5. Serum Preparation and Enzyme-Linked Immunosorbent Assay (ELISA) for OVA-Specific IgE in Serum

Blood samples were collected via cardiac puncture and centrifuged at 3000× *g* for 15 min at 4 °C to obtain serum. OVA-specific IgE levels were quantified using a commercial ELISA kit (LEGEND MAX^TM^, BioLegend, San Diego, CA, USA) following the manufacturer’s protocol.

### 2.6. Hematoxylin and Eosin (H&E) Staining

Enucleated eyes were fixed, paraffin-embedded, and sectioned. Tissue sections were stained with H&E (Abcam, Cambridge, UK) to evaluate inflammatory cell infiltration and vascular changes in the conjunctiva. Quantification of PMN cells and hemangiectasis was performed using ImageJ software.

### 2.7. Toluidine Blue Staining

Mast cell distribution and degranulation were assessed using toluidine blue staining. Sections were stained with 1% toluidine blue (Daejeon, Republic of Korea), differentiated, and mounted. Degranulated mast cells were identified by metachromatic granules and quantified using ImageJ.

### 2.8. Immunofluorescence Staining

Paraffin-embedded tissues were sectioned, deparaffinized, rehydrated, and subjected to antigen retrieval. After blocking with 0.1% BSA containing normal goat serum, sections were incubated overnight at 4 °C with primary antibodies (Supplementary Table S1). Following PBS washes, sections were incubated with the corresponding fluorophore-conjugated secondary antibodies (Supplementary Table S1) for 1 h at room temperature in the dark, counterstained with DAPI, and mounted.

After immunostaining, seven samples (*n* = 7) from each group were analyzed. Representative fields were randomly selected from the tissue sections for analysis, and the same selection criteria were consistently applied across all experimental groups. For quantitative immunofluorescence analysis, identical image-acquisition and thresholding criteria were applied across all experimental groups to minimize variation in fluorescence-based quantification. Fluorescence images were acquired using a confocal microscope (Carl Zeiss, Inc., Jena, Germany).

### 2.9. Periodic Acid–Schiff (PAS) Staining

For PAS staining, tissue sections were treated with 1% PAS solution and Schiff’s reagent (Sigma-Aldrich, Burlington, MA, USA), followed by Harris hematoxylin counterstaining. The sections were then washed, dehydrated, cleared, and mounted for microscopic evaluation.

### 2.10. Statistical Analyses

Statistical analyses were performed using GraphPad Prism (version 5.01), and image quantification was conducted with ImageJ (version 1.62f). Data are presented as mean ± SEM. One-way ANOVA with Tukey’s test was used for multiple-group comparisons, and Bartlett’s test assessed variance homogeneity. A *p*-value < 0.05 was considered significant.

## 3. Results

### 3.1. OLO Attenuates Clinical Allergic Symptoms in an OVA-Induced AED Mouse Model

The AED mouse model was successfully established through OVA sensitization and challenge, as evidenced by characteristic clinical manifestations. Mice in the OVA group exhibited pronounced ocular symptoms, including conjunctival hyperemia, chemosis, eyelid edema, and increased mucoid discharge, confirming the induction of allergic inflammation.

In contrast, the normal group displayed no observable pathological features, indicating the absence of ocular allergic responses. Importantly, the OVA + OLO group showed markedly reduced redness, swelling, and secretion compared with the OVA group (Figure 2B,D).

### 3.2. OLO Reduces Allergen-Induced Periocular Scratching Behavior in an OVA-Induced AED Mouse Model

Periocular scratching behavior was used as a behavioral indicator of allergen-induced ocular discomfort in the OVA-induced AED mouse model. Mice in the OVA group exhibited a significant increase in periocular scratching frequency compared with the normal group, indicating successful induction of allergic ocular responses.

Correspondingly, the OVA + OLO group exhibited a marked reduction in periocular scratching behavior compared with the OVA group, indicating attenuation of allergen-induced ocular discomfort-related behavioral responses (Figure 2E).

### 3.3. OLO Suppresses Serum IgE Levels in an OVA-Induced AED Mouse Model

To evaluate the allergic response, OVA-specific IgE levels were measured in serum using ELISA. A significant elevation in IgE levels was observed in the OVA group compared to the normal group, consistent with the induction of an IgE-associated allergic response.

Moreover, the OVA + OLO group exhibited a marked reduction in serum IgE levels compared with the OVA group, suggesting an attenuation of the allergic response following OLO treatment (Figure 2F).

### 3.4. OLO Inhibits Conjunctival Mast Cell Degranulation in an OVA-Induced AED Mouse Model

Toluidine blue staining was used to evaluate mast cell activity in the conjunctiva following OVA challenge. The OVA group triggered pronounced mast cell degranulation. This response was largely absent under normal conditions, where mast cells remained intact with minimal degranulation.

**Figure 3:**
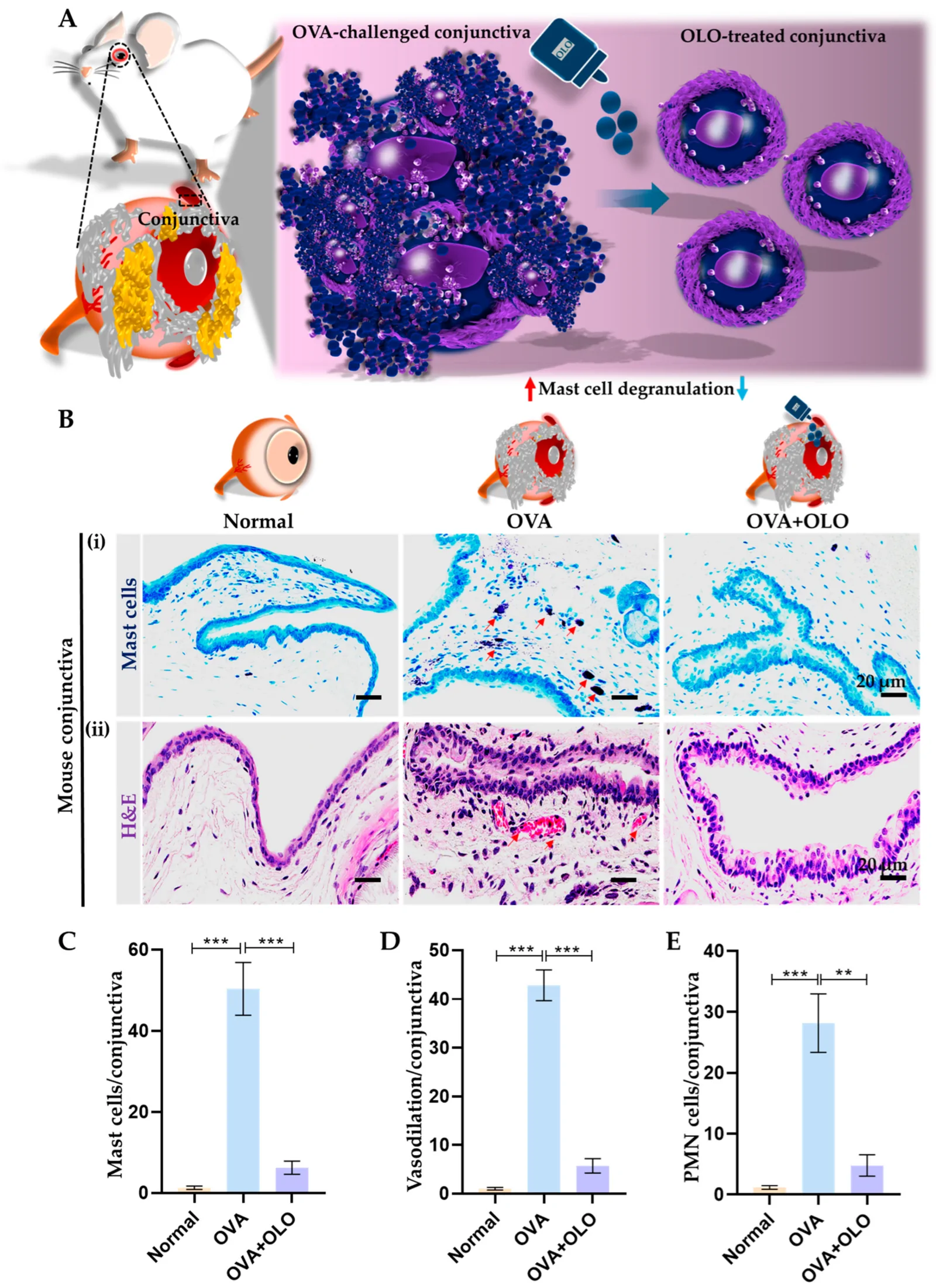
OLO attenuates conjunctival mast cell degranulation, PMN cell infiltration, and vasodilation in an OVA-induced AED mouse model: (**A**) Schematic illustration of mast cell degranulation in the OVA-challenged and OLO-treated conjunctiva. In the OVA-challenged conjunctiva, the conjunctival tissue exhibits marked mast cell degranulation, characterized by granule release. In contrast, OLO treatment reduces mast cell degranulation, resulting in fewer degranulated mast cells. (**B**) (**i**) Toluidine blue staining to demonstrate mast cell degranulation in the conjunctiva. (**ii**) H&E staining to demonstrate the PMN and vasodilation in the conjunctiva. Quantification of (**C**) mast cell degranulation, (**D**) vasodilation, and (**E**) PMN cell infiltration in the conjunctiva. In (**B**), red arrows indicate (**i**) mast cell degranulation and (**ii**) conjunctival vasodilation in the OVA group. Mean ± SEM (*n* = 7). One-way ANOVA followed by Tukey’s post hoc test. ** *p*  <  0.01; *** *p*  <  0.001.

The OVA + OLO group showed markedly reduced mast cell degranulation compared with the OVA group (Figure 3B(i) and Figure 3C, respectively).

### 3.5. OLO Attenuates Conjunctival Vasodilation and PMN Cell Infiltration in an OVA-Induced AED Mouse Model

Histological analysis of conjunctival tissues revealed no notable vasodilation or PMN cell infiltration in the normal group. In contrast, the OVA group exhibited marked conjunctival vasodilation (hemangiectasis) and substantial infiltration of PMN cells.

Correspondingly, the OVA + OLO group showed significantly reduced vascular dilation and PMN cell infiltration compared with the OVA group (Figure 3B(ii) and Figure 3D,E, respectively).

### 3.6. OLO Reduces Neuropeptide-Associated Marker Immunoreactivity in the Conjunctiva in an OVA-Induced AED Mouse Model

Next, immunofluorescence staining was performed to evaluate SP, TRPV1, and CGRP immunoreactivity in conjunctival tissues. In the OVA group, immunoreactivity for SP, TRPV1, and CGRP was markedly increased in the conjunctiva compared with the normal group, indicating increased expression of these markers during allergic inflammation.

**Figure 4:**
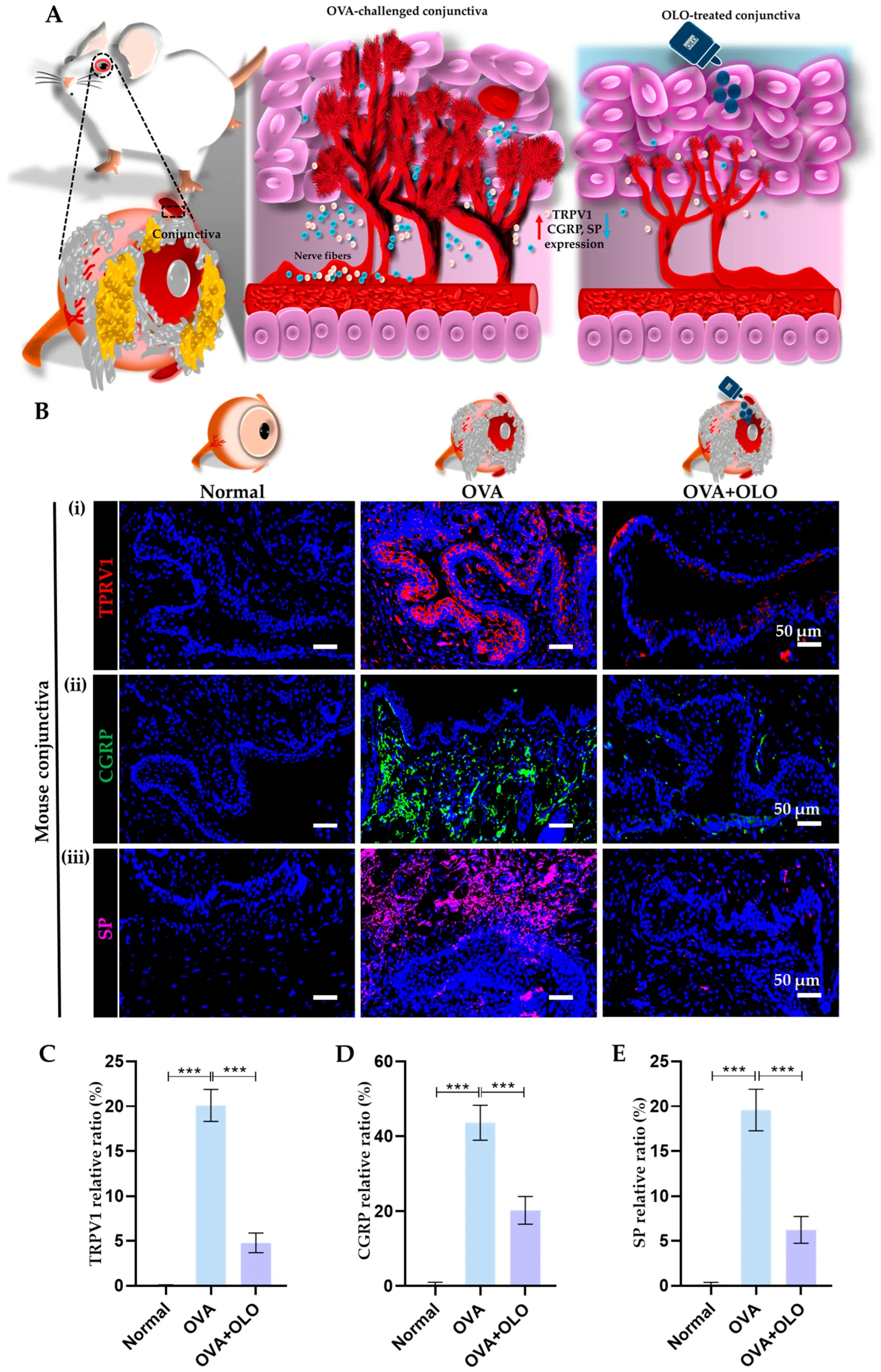
Effect of OLO on neuropeptide-associated marker expression in an OVA-induced AED mouse model: (**A**) Conceptual illustration of neuropeptide-associated marker expression within the conjunctiva in an OVA-induced AED mouse model and the effect of OLO. OVA challenge induces increased expression of TRPV1, SP, and CGRP in the conjunctiva. These changes indicate altered expression of neuropeptide-associated markers in AED. In contrast, OLO treatment attenuates these changes, as reflected by reduced expression of TRPV1, SP, and CGRP in the conjunctiva. (**B**) Immunofluorescence staining for (**i**) TRPV1, (**ii**) CGRP, and (**iii**) SP in the conjunctiva. Nuclei were counterstained with DAPI for visualization. Quantification of (**C**) TRPV1, (**D**) CGRP, and (**E**) SP in the conjunctiva. Immunopositivity in the conjunctival tissue was quantified as the percentage of immunopositive cells relative to the total number of DAPI-positive cells. Mean ± SEM (*n* = 7). One-way ANOVA followed by Tukey’s post hoc test. *** *p*  <  0.001.

In contrast, immunoreactivity for SP, TRPV1, and CGRP was markedly reduced in the OVA + OLO group compared with the OVA group, indicating that OLO attenuated the increased expression of these markers in the conjunctiva (Figure 4B(i–iii) and Figure 4C–E, respectively).

### 3.7. OLO Attenuates Conjunctival Macrophage Infiltration and iNOS Immunoreactivity in an OVA-Induced AED Mouse Model

Immunofluorescence staining was performed to evaluate macrophage infiltration and inducible nitric oxide synthase (iNOS) expression in the conjunctival tissue. In the OVA group, a marked increase in F4/80-positive cells was observed, indicating significant macrophage accumulation in response to allergic inflammation, whereas the normal group exhibited minimal F4/80 expression.

In parallel, iNOS staining demonstrated a substantial increase in iNOS-positive cells in the OVA group, indicating increased iNOS expression during allergic inflammation. In contrast, the normal group showed negligible iNOS expression.

**Figure 5:**
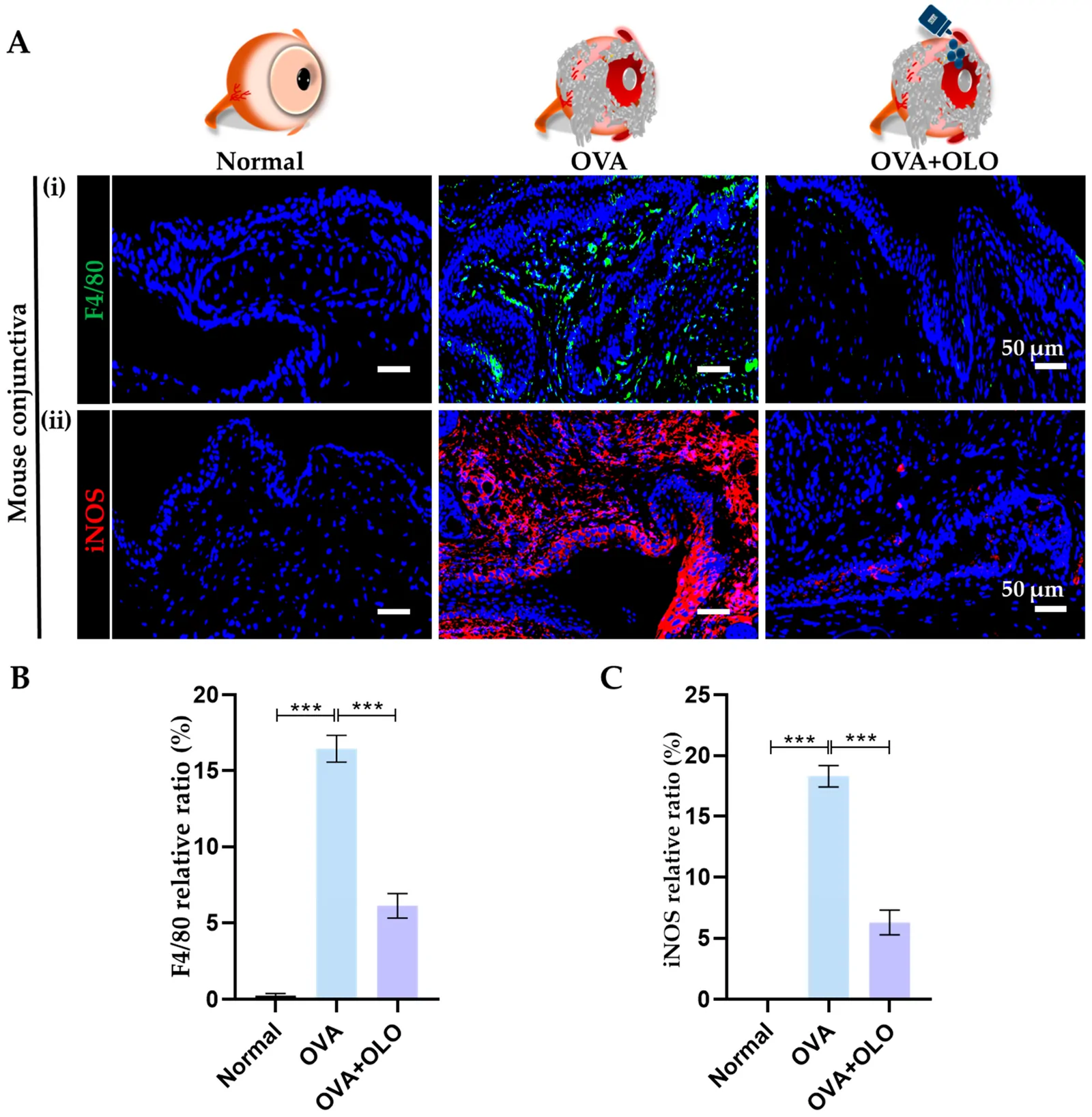
Effects of OLO on conjunctival macrophage infiltration and iNOS expression in an OVA-induced AED mouse model: (**A**) Immunofluorescence staining for (**i**) F4/80 and (**ii**) iNOS in the conjunctiva. Nuclei were counterstained with DAPI for visualization. Quantification of (**B**) F4/80 and (**C**) iNOS. Immunopositivity in the conjunctival tissue was quantified as the percentage of immunopositive cells relative to the total number of DAPI-positive cells. Mean ± SEM (*n* = 7). One-way ANOVA followed by Tukey’s post hoc test. *** *p*  <  0.001.

Notably, treatment with OLO significantly reduced both F4/80-positive macrophage infiltration and iNOS expression compared with the OVA group (Figure 5A(i,ii) and Figure 5B,C, respectively).

### 3.8. OLO Reduces Conjunctival Pro-Inflammatory Cytokine Immunoreactivity in an OVA-Induced AED Mouse Model

Immunofluorescence staining demonstrated markedly increased immunoreactivity for pro-inflammatory cytokines, including IL-6, TNF-α, and IL-1β, in the conjunctival tissues of the OVA group.

**Figure 6:**
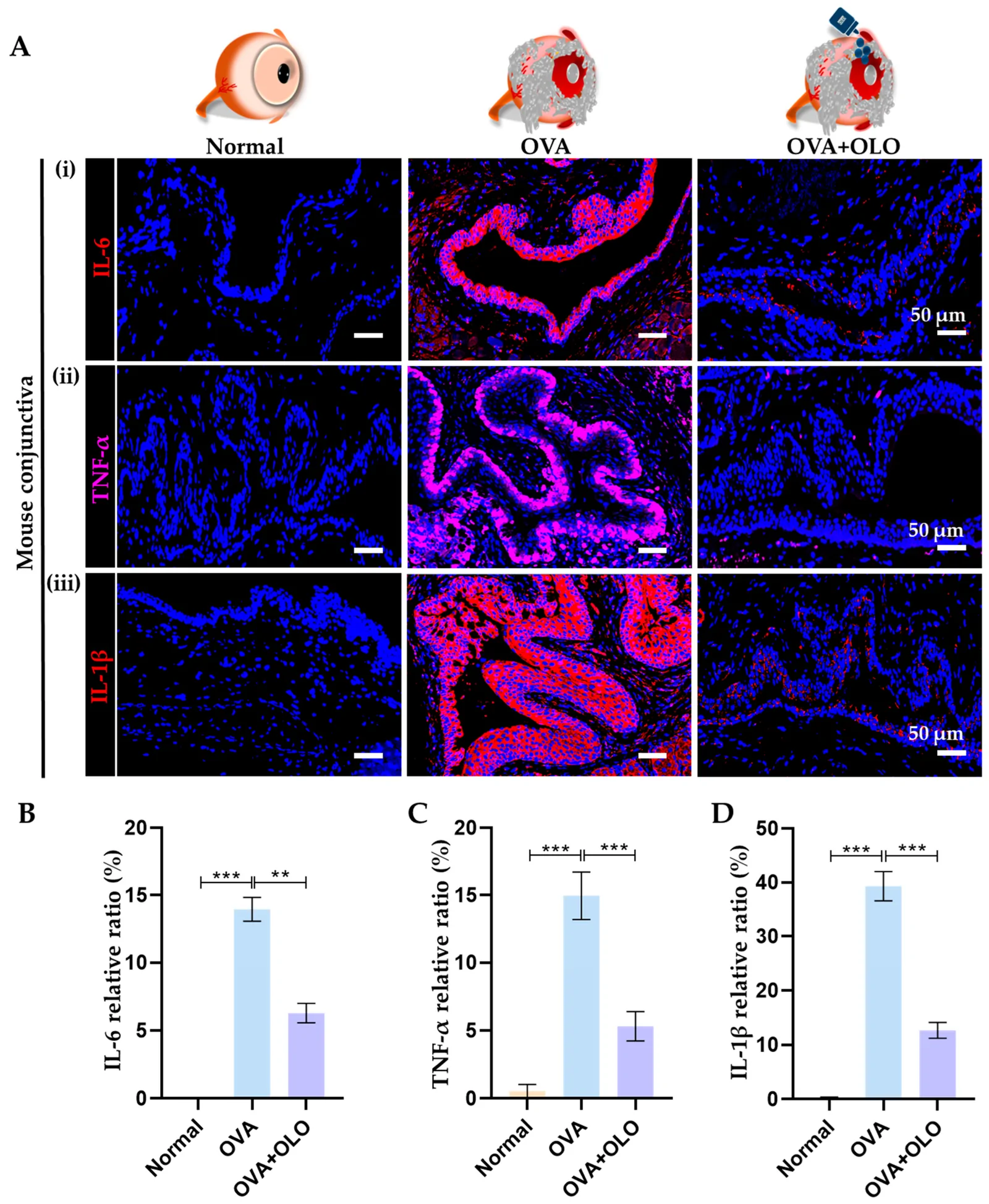
Effects of OLO on conjunctival pro-inflammatory cytokine immunoreactivity in an OVA-induced AED mouse model: (**A**) Immunofluorescence staining for (**i**) IL-6, (**ii**) TNF-α, and (**iii**) IL-1β in the conjunctiva. Nuclei were counterstained with DAPI for visualization. Quantification of (**B**) IL-6, (**C**) TNF-α, and (**D**) IL-1β in the conjunctiva. Immunopositivity in the conjunctival tissue was quantified as the percentage of immunopositive cells relative to the total number of DAPI-positive cells. Mean ± SEM (*n* = 7). One-way ANOVA followed by Tukey’s post hoc test. ** *p*  <  0.01; *** *p*  <  0.001.

In contrast, the normal group exhibited minimal or undetectable immunoreactivity for these cytokines. Likewise, in the OVA + OLO group, the immunoreactivity of IL-6, TNF-α, and IL-1β was markedly reduced, indicating that OLO reduced the local immunoreactivity of these pro-inflammatory markers in the allergic conjunctiva (Figure 6A(i–iii) and Figure 6B–D, respectively).

### 3.9. OLO Reduces Conjunctival Th2-Associated Cytokine Immunoreactivity in an OVA-Induced AED Mouse Model

Immunofluorescence staining revealed markedly increased immunoreactivity for the Th2-associated cytokines IL-4 and IL-5 in the conjunctiva of the OVA group compared with the normal group.

**Figure 7:**
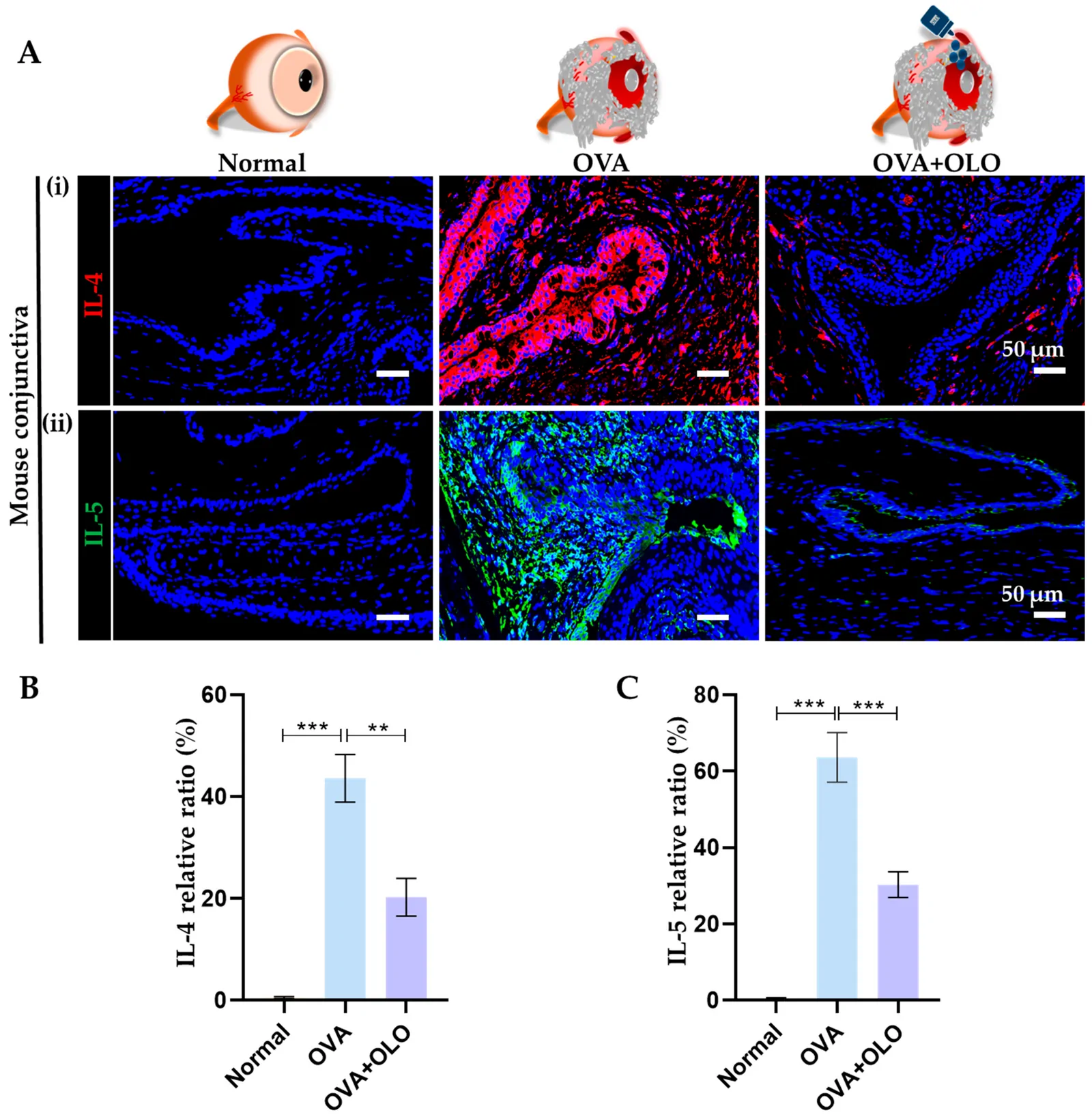
Effect of OLO on conjunctival Th2-associated cytokine immunoreactivity in an OVA-induced AED mouse model: (**A**) Immunofluorescence staining for (**i**) IL-4 and (**ii**) IL-5 in the conjunctiva. Nuclei were counterstained with DAPI for visualization. Quantification of (**B**) IL-4 and (**C**) IL-5 in the conjunctiva. Immunopositivity in the conjunctival tissue was quantified as the percentage of immunopositive cells relative to the total number of DAPI-positive cells. Mean ± SEM (*n* = 7). One-way ANOVA followed by Tukey’s post hoc test. ** *p*  <  0.01; *** *p*  <  0.001.

In contrast, no detectable immunofluorescence for IL-4 and IL-5 was observed in the normal group. Following OLO treatment, immunofluorescence for IL-4 and IL-5 was significantly reduced in the OVA + OLO group compared with the OVA group (Figure 7A(i,ii) and Figure 7B,C, respectively).

### 3.10. OLO Preserves Goblet Cell Density in an OVA-Induced AED Mouse Model

PAS staining was performed to evaluate goblet cell density in the conjunctiva. Goblet cell density was markedly reduced in the OVA group compared with the normal group.

**Figure 8:**
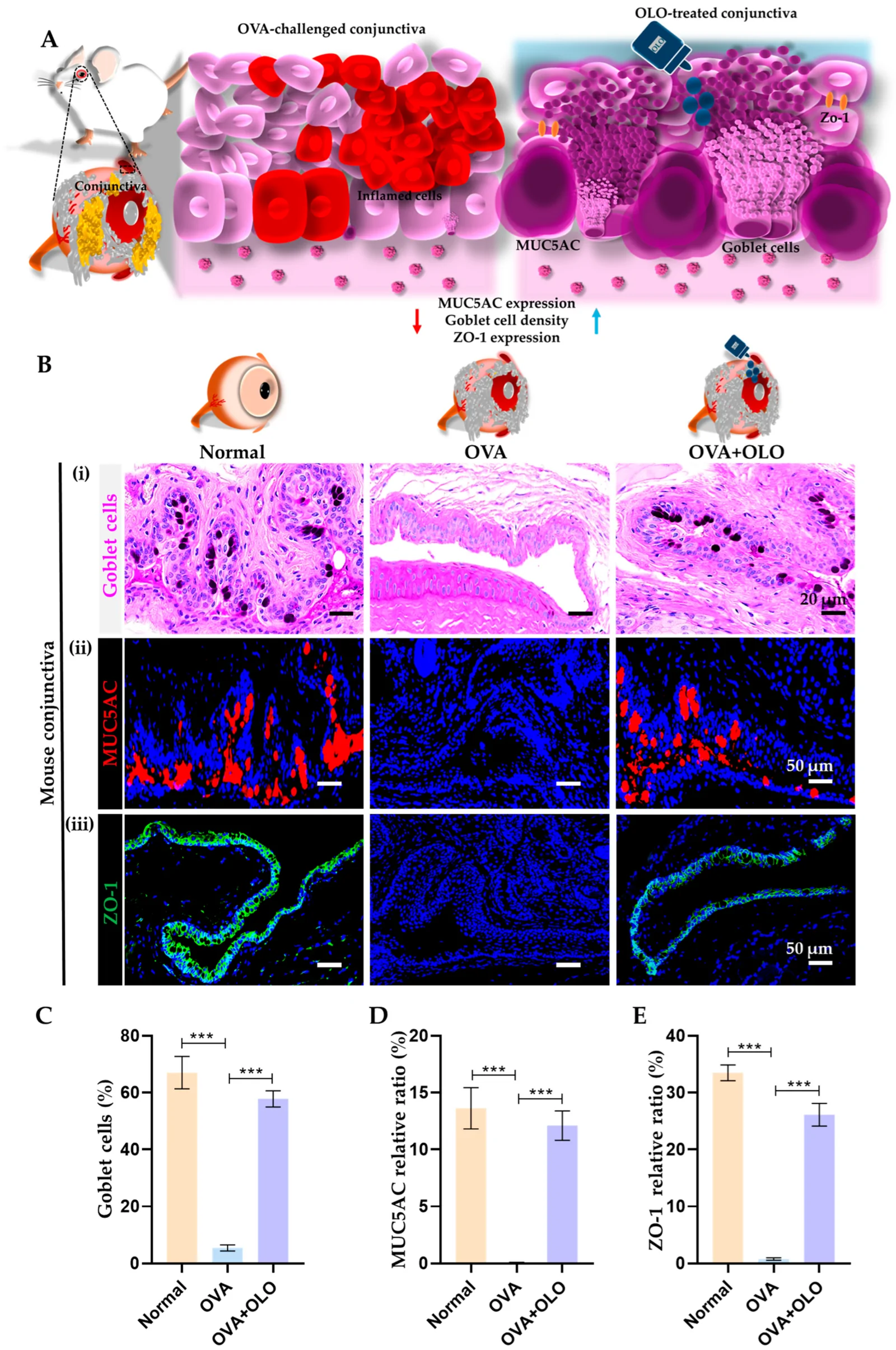
Effect of OLO on conjunctival epithelial–mucosal changes in an OVA-induced AED mouse model: (**A**) Schematic representation of conjunctival epithelial–mucosal changes in an OVA-induced AED mouse model and the protective effects of OLO. OVA challenge leads to a reduction in goblet cell density and decreased expression of MUC5AC and ZO-1. In contrast, OLO treatment preserves goblet cell populations and MUC5AC and ZO-1 expression, indicating preservation of these epithelial–mucosal features. (**B**) PAS staining for (**i**) goblet cells and immunofluorescence staining for (**ii**) MUC5AC and (**iii**) ZO-1 in the conjunctiva. In (**B**) (**ii**,**iii**), nuclei were counterstained with DAPI for visualization, and immunopositivity in the conjunctival tissue was quantified as the percentage of immunopositive cells relative to the total number of DAPI-positive cells. Quantification of (**C**) goblet cells, (**D**) MUC5AC, and (**E**) ZO-1 in the conjunctiva. Mean ± SEM (*n* = 7). One-way ANOVA followed by Tukey’s post hoc test. *** *p*  <  0.001.

In contrast, the normal group maintained a stable goblet cell population. Goblet cell density was significantly higher in the OVA + OLO group compared with the OVA group (Figure 8B(i) and Figure 8C, respectively).

### 3.11. OLO Increases Conjunctival MUC5AC Expression in an OVA-Induced AED Mouse Model

Further, immunofluorescence staining for mucin 5AC (MUC5AC), a major secretory mucin of the conjunctiva, was performed to assess MUC5AC expression in conjunctival tissue. The OVA group mice exhibited a marked reduction in MUC5AC expression.

In contrast, the normal group showed strong MUC5AC expression. The OVA + OLO group exhibited significantly higher MUC5AC expression compared with the OVA group (Figure 8B(ii) and Figure 8D, respectively).

### 3.12. OLO Preserved ZO-1 Expression in an OVA-Induced AED Mouse Model

Immunofluorescence staining for zonula occludens-1 (ZO-1), a key tight junction protein, was performed to evaluate ZO-1 expression in the conjunctival epithelium. The OVA group exhibited markedly reduced ZO-1 expression compared with the normal group.

In contrast, the normal group showed strong and continuous ZO-1 expression. Notably, the OVA + OLO group exhibited significantly higher ZO-1 expression compared with the OVA group (Figure 8B(iii) and Figure 8E, respectively).

### 3.13. OLO Mitigates Conjunctival Apoptosis in an OVA-Induced AED Mouse Model

TUNEL staining was conducted to evaluate apoptotic activity within the conjunctival tissue. A substantial increase in TUNEL-positive cells was observed in the OVA group compared with the normal group, indicating increased apoptosis following allergen exposure. The normal group exhibited no evidence of apoptotic cell death.

**Figure 9:**
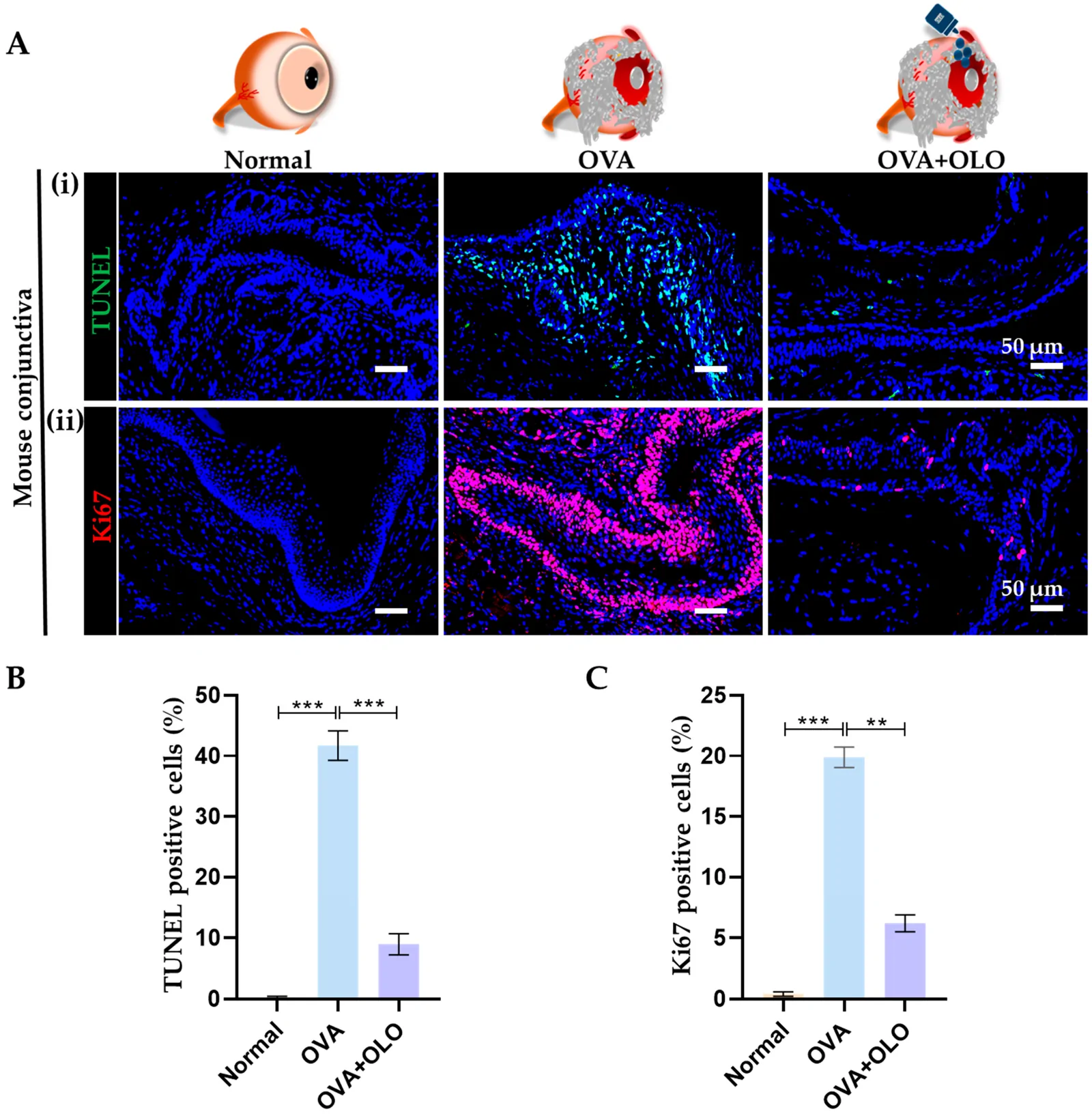
Effect of OLO on conjunctival apoptosis and epithelial cell proliferation in an OVA-induced AED mouse model: (**A**) (**i**) TUNEL staining and (**ii**) immunofluorescence staining for Ki67 in the conjunctiva. Quantification of (**B**) TUNEL and (**C**) Ki67-positive cells in the conjunctiva. Nuclei were counterstained with DAPI for visualization. Immunopositivity in the conjunctival tissue was quantified as the percentage of immunopositive cells relative to the total number of DAPI-positive cells. Mean ± SEM (*n* = 7). One-way ANOVA followed by Tukey’s post hoc test. *** *p* < 0.001.

The OVA + OLO group showed significantly fewer TUNEL-positive cells than the OVA group, indicating reduced allergen-induced apoptosis (Figure 9A(i) and Figure 9B, respectively).

### 3.14. OLO Diminishes Conjunctival Cellular Proliferation in an OVA-Induced AED Mouse Model

Ki67 immunostaining was performed to assess dysregulated epithelial cell proliferation in the conjunctiva. A marked increase in Ki67-positive cells was observed in the conjunctival epithelium of the OVA group, whereas no Ki67 expression was detected in the normal group.

Interestingly, the OVA + OLO group exhibited a significant reduction in Ki67-positive cells compared with the OVA group, indicating that OLO reduced conjunctival epithelial cell proliferation (Figure 9A(ii) and Figure 9C, respectively).

## 4. Discussion

This study provides an integrated assessment of the effects of OLO on conjunctival immune–inflammatory responses, neuropeptide-associated marker expression, and epithelial–mucosal changes in an OVA-induced mouse model of AED. Although the anti-allergic and anti-inflammatory effects of OLO are well established, this study extends these findings by examining immune–inflammatory, neuropeptide-associated, and epithelial–mucosal responses simultaneously within a single experimental model. AED is a multifactorial disorder involving IgE-mediated hypersensitivity, mast cell activation, inflammatory cell infiltration, neuropeptide-associated processes, and epithelial–mucosal changes [8,27,28,29]. OLO is clinically established as a histamine H1 receptor antagonist and mast cell stabilizer, but its broader effects on immune–inflammatory regulation, neuropeptide-associated responses, and epithelial–mucosal responses remain incompletely understood. Our findings demonstrate that OLO not only alleviates classical allergic inflammatory responses but also reduces the expression of neuropeptide-associated markers and preserves ZO-1 expression and goblet cell populations.

Our results are consistent with previous observations of immune–inflammatory changes in AED. OVA sensitization and challenge led to elevated serum IgE, robust mast cell degranulation, infiltration of PMNs and F4/80-positive macrophages, and increased conjunctival immunoreactivity for pro-inflammatory and Th2-associated cytokine markers, including IL-4, IL-5, IL-6, TNF-α, and IL-1β [18,25,30,31,32,33,34,35]. In conjunctival tissues, OVA challenge prominently increased mast cell degranulation (Figure 3A) [7,36], consistent with IgE-mediated mast cell responses associated with both early- and late-phase allergic inflammation [37,38]. In contrast, OLO treatment significantly downregulated mast cell degranulation, reduced IgE levels, attenuated inflammatory cell infiltration, and reduced the immunoreactivity of several inflammatory cytokine markers, reflecting suppression of key allergic effector responses [15,39,40]. The reduction in serum OVA-specific IgE following topical OLO treatment represents an additional finding of the present study. However, because systemic OLO exposure and additional systemic immunological parameters were not evaluated, the basis of this reduction cannot be definitively determined. Thus, this finding should be interpreted as an association between topical OLO treatment and reduced serum OVA-specific IgE levels rather than as evidence of a direct effect on systemic IgE responses.

Beyond classical immune pathways, neuropeptide-associated inflammation has been increasingly recognized as a critical component of AED, contributing to ocular sensory and allergic responses [41]. Sensory nerve-derived mediators, including SP and CGRP, have been implicated in vascular dilation, immune cell recruitment, tissue remodeling, and sensory responses, thereby contributing to the development and progression of AED [42,43,44,45,46,47]. TRPV1, a key sensory signaling molecule expressed on conjunctival sensory nerves, is also involved in allergic and sensory responses [48,49]. During allergic ocular inflammation, sensory nerves may become sensitized, potentially enhancing sensations such as itch and irritation [50]. Although functional interactions among TRPV1, SP, and CGRP have been reported [12,51,52,53,54], the present study assessed their expression rather than neuronal activation.

In our study, OVA-challenged mice exhibited marked upregulation of TRPV1, SP, and CGRP in the conjunctiva (Figure 4A), indicating altered expression of neuropeptide-associated markers during allergic ocular inflammation. Notably, OLO treatment significantly reduced the expression of these markers. Together, these findings suggest that changes in neuropeptide-associated marker expression accompany the inflammatory response in this model and are modified following OLO treatment. These coordinated changes further characterize the ocular response to allergen challenge and the effects observed following OLO treatment.

In parallel, OVA-challenged mice exhibited significantly increased periocular scratching behavior, which was markedly reduced following OLO treatment (Figure 2C,E). In humans, AED is typically characterized by persistent itching that leads to frequent eye rubbing, representing a hallmark clinical symptom [11]. Thus, the reduction in scratching behavior following OLO treatment may indicate attenuation of ocular discomfort-related behavioral responses in this experimental model.

Epithelial changes represent another important aspect of AED. The conjunctival epithelium forms a selective barrier that regulates allergen penetration, maintains mucosal homeostasis, and supports goblet cell populations [55]. Previous studies have highlighted that allergic inflammation impairs tight junction proteins, alters epithelial turnover, and reduces goblet cell density, thereby contributing to persistent inflammation and mucosal dysfunction [8,56,57]. In addition, a clinical study comparing patients with seasonal allergic conjunctivitis with healthy controls demonstrated reduced expression of epithelial junctional proteins, further supporting epithelial barrier impairment in AED [57]. In the present study, OVA challenge was associated with reduced ZO-1 expression (Figure 8A) and increased numbers of Ki67- and TUNEL-positive cells in the conjunctival epithelium. OLO treatment preserved ZO-1 expression and reduced both Ki67- and TUNEL-positive cell populations. These findings suggest that OLO may modulate OVA-associated epithelial changes, including alterations in ZO-1 expression, cellular proliferative activity, and apoptosis.

Furthermore, conjunctival goblet cells are the principal source of MUC5AC, the major gel-forming mucin of the tear film that contributes to ocular-surface lubrication and mucosal protection [58]. In AED, allergic inflammation may alter goblet cell populations and MUC5AC expression, leading to changes in the conjunctival mucosal environment. In the present study, OVA challenge significantly reduced both goblet cell density and MUC5AC expression. Importantly, OLO treatment preserved the goblet cell population and increased MUC5AC expression (Figure 8A). Clinically, OLO treatment has also been associated with increased conjunctival goblet cell density and improvement of ocular surface parameters in patients with allergic conjunctivitis, supporting its effects on conjunctival epithelial–mucosal features [59].

Taken together, our findings demonstrate that OLO exerts multifaceted protective effects in AED by modulating classical immune responses, altering neuropeptide-associated marker expression, and preserving ZO-1 expression and goblet cell populations [3,8,12].

Clinically, OLO is a dual-action antihistamine and mast-cell stabilizer widely used for allergic conjunctivitis. Other topical dual-action agents, including ketotifen, azelastine, epinastine, and alcaftadine, also effectively reduce allergic symptoms through antihistamine and mast-cell stabilizing activities [10,60]. Anti-inflammatory therapies, such as topical corticosteroids, may provide stronger suppression of ocular inflammation in severe or refractory disease but have greater safety considerations [61]. Although our findings suggest that OLO may additionally modulate inflammatory, neuropeptide-associated, epithelial, and mucin-associated responses, these findings do not establish superiority over other topical therapies because no direct comparator was included. Direct head-to-head studies will be needed to determine whether these broader effects provide a clinically meaningful advantage.

Several limitations should be acknowledged. First, the OVA-induced model represents an acute allergic response and may not fully recapitulate the chronic or recurrent nature of human seasonal or perennial AED. Although this model reproduces several key features of allergic inflammation, it does not fully capture the prolonged and recurrent allergen exposure characteristic of chronic human disease. In addition, differences between the mouse and human ocular surfaces may limit direct clinical translation. OLO was administered during the OVA challenge period rather than after clinical establishment of AED, and only a single dose and short treatment period were evaluated; therefore, effects on established disease, dose-dependent responses, and long-term outcomes remain undetermined. The absence of a vehicle control, OLO-only group, and direct comparator also limits assessment of OLO-specific effects and comparison with other therapies.

Second, the neuropeptide-related findings were based on immunofluorescence assessment of TRPV1, SP, and CGRP expression and therefore do not establish neuronal activation. Similarly, cytokine and neuropeptide-associated immunofluorescence provides evidence of marker expression but does not establish total protein abundance. Eosinophils were not specifically characterized. Although OLO reduced serum OVA-specific IgE, the underlying mechanism and potential contribution of systemic OLO exposure were not investigated.

Finally, changes in ZO-1, Ki67, TUNEL, and MUC5AC were observed following the OVA challenge and OLO treatment; however, epithelial barrier function, cytoprotection, and mucin secretion were not directly evaluated. Objective tear production, fluorescein staining, and quantitative hyperemia were also not assessed. Future studies incorporating post-establishment treatment, dose–response and long-term assessments, functional neuropeptide and barrier analyses, and appropriate comparators are warranted.

## 5. Conclusions

In conclusion, this study shows that OLO exerts multifaceted protective effects on the ocular surface in an OVA-induced AED mouse model. OLO treatment was associated with reduced inflammatory responses, decreased expression of neuropeptide-associated markers, and changes in conjunctival epithelial–mucosal features. Collectively, these findings suggest that OLO provides protective effects during OVA-induced allergic inflammation and support its potential benefits in this experimental model of AED. Future studies are warranted to further clarify the mechanisms underlying these effects and evaluate the long-term effects of OLO.

## Data Availability

All datasets used and/or analyzed during the current study are available from the corresponding author upon reasonable request.

## Supplementary Materials

The following supporting information can be downloaded at https://www.mdpi.com/article/10.3390/bioengineering13091060/s1, Table S1: Antibody information for immunofluorescence staining.

## Abbreviations

OLO	Olopatadine
AED	Allergic eye disease
OVA	Ovalbumin
IgE	Immunoglobulin E
PMNs	Polymorphonuclear leukocytes
IL	Interleukin
TNF-α	Tumor necrosis factor-α
IL-1β	Interleukin-1β
SP	Substance P
CGRP	Calcitonin gene-related peptide
TRPV1	Transient receptor potential vanilloid 1
ZO-1	Zonula occludens-1
PAS	Periodic acid–Schiff
H&E	Hematoxylin and eosin
IACUC	Institutional Animal Care and Use Committee
MUC5AC	Mucin 5AC
TUNEL	Terminal deoxynucleotidyl transferase dUTP nick end labeling
ELISA	Enzyme-linked immunosorbent assay
Th2	T helper 2
IP	Intraperitoneal injection
ALUM	Aluminum hydroxide
PT	Pertussis toxin
