# Supplementary material for: Olopatadine Exerts Multifaceted Protective Effects in an Ovalbumin-Induced Allergic Eye Disease Mouse Model
Source: Bioengineering (Basel). 2026 Sep 12;13(9):1060. doi: 10.3390/bioengineering13091060 (PMC13603604; doi:10.3390/bioengineering13091060)
Supplement: Supplementary file 1 [file bioengineering-13-01060-s001.zip › bioengineering-4482461-supplementary.pdf]

Table S1: Antibody information for immunofluorescence staining.

No.	Target (Antibody) for Western Blot	Catalog number	Application	Dilution	Supplier (Official Name; Headquarters City/State/Country)
1	IL-4	sc-53084	Primary	1:200	Santa Cruz Biotechnology, Inc.; Santa Cruz, CA, USA
2	IL-5	sc-398334	Primary	1:100	Santa Cruz Biotechnology, Inc.; Santa Cruz, CA, USA
3	TNF-α	3707	Primary	1:200	Santa Cruz Biotechnology, Inc.; Santa Cruz, CA, USA
4	IL-1β	ab315084	Primary	1:100	Abcam, Inc.; Cambridge, UK
5	F4/80	ab6640	Primary	1:200	Abcam, Inc.; Cambridge, UK
6	IL-6	ab9324	Primary	1:100	Abcam, Inc.; Cambridge, UK
7	TRPV1	ab305299	Primary	1:100	Abcam, Inc.; Cambridge, UK
8	CGRP	ab47027	Primary	1:200	Abcam, Inc.; Cambridge, UK
9	MUC5AC	ab3649	Primary	1:200	Abcam, Inc.; Cambridge, UK
10	ZO-1	617300	Primary	1:200	Invitrogen, Inc.; Cambridge, UK
11	Ki67	ab15580	Primary	1:200	Abcam, Inc.; Cambridge, UK
12	SP	ab14184	Primary	1:50	Abcam, Inc.; Cambridge, UK
13	iNOS	ab15323	Primary	1:200	Abcam, Inc.; Cambridge, UK
14	Alexa Fluor 488	A11008	Secondary	1:400	Thermo Fisher Scientific Inc.; W altham, MA, USA
15	Alexa Fluor 555	A21424	Secondary	1:400	Thermo Fisher Scientific Inc.; W altham, MA, USA
16	Alexa Fluor 568	A10042	Secondary	1:400	Thermo Fisher Scientific Inc.; W altham, MA, USA
17	Alexa Fluor 488	A11029	Secondary	1:400	Thermo Fisher Scientific Inc.; W altham, MA, USA
